# AI-Driven Human Pose Estimation Reveals Distinct Gait Patterns in Older Adults with Physio-Cognitive Decline

**DOI:** 10.1093/geroni/igaf122.3701

**Published:** 2025-12-31

**Authors:** Zilong Zhang, Yiwen Xing, Lina Ma

**Affiliations:** Xuanwu Hospital, Capital Medical University, Beijing, Beijing, China (People’s Republic); Xuanwu Hospital, Capital Medical University, Beijing, Beijing, China (People’s Republic); Xuanwu Hospital, Capital Medical University, Beijing, Beijing, China (People’s Republic)

## Abstract

Physio-Cognitive Decline Syndrome (PCDS) is a geriatric condition characterized by coexisting Mobility Impairment No Disability (MIND) and Cognitive Impairment No Dementia (CIND). Gait dysfunction, which commonly occurs in older adults, can serve as an indicator of underlying pathological and functional states. This study employed an AI-based human pose estimation algorithm to analyze gait patterns and quantitatively extract gait parameters in older adults with PCDS. A total of 755 community-dwelling adults aged 65 years or older were included in this study. Participants performed a 4-meter walking test while being recorded via intelligent software; gait information was extracted from 17 key skeletal points using pose estimation technology, and an action recognition algorithm was applied to analyze and predict gait abnormalities. After adjusting for sociodemographic factors, lifestyle, chronic diseases, polypharmacy, nutrition, and psychological conditions, gait dysfunction identified by the pose estimation algorithm was associated with an increased risk of PCDS. Higher connection velocity, joint point acceleration, and joint point angular velocity were protective against PCDS. Stride length showed a positive correlation with immediate and delayed recall memory among PCDS participants, even after adjusting for age and sex. These findings demonstrate that AI-driven pose estimation offers valuable clinical insight into identifying PCDS-related gait impairments, highlighting its potential as a tool for early screening and mobility-cognitive integration assessment in community-dwelling older adults.

